# Effect of Chaihu Guizhi granules on clinical parameters and vascular endothelial function in patients with stable angina pectoris: A randomized, double-blind, placebo-controlled trial

**DOI:** 10.1097/MD.0000000000045631

**Published:** 2025-11-07

**Authors:** Yu Cao, Sihui Wang, Menghua He, Yan Shen

**Affiliations:** a Department of Geriatrics, Yueyang Hospital of Integrative Medicine, Shanghai University of Traditional Chinese Medicine, Shanghai, China; b Shanghai University of Traditional Chinese Medicine, Shanghai, China; c Department of Cardiology, Yueyang Hospital of Integrative Medicine, Shanghai University of Traditional Chinese Medicine, Shanghai, China.

**Keywords:** Chaihu Guizhi decoction, coronary atherosclerotic heart disease, herbal medicine, integrated traditional Chinese and Western medicine, vascular endothelium

## Abstract

**Background:**

Coronary artery disease (CAD) is a major cause of health problems and deaths worldwide. Endothelial function is an independent predictor of CAD. Chinese herbal medicine has been proven to effectively improve the clinical symptoms and prognosis of patients with CAD. The purpose of this study was to evaluate the efficacy and safety of Chaihu Guizhi granules in patients with stable angina pectoris (SAP), explore the possible mechanism through which Chaihu Guizhi formula (CHGZ) improves vascular endothelial function, and provide evidence-based medical support for the rational clinical use of CHGZ.

**Methods:**

In this randomized, double-blind, placebo-controlled study, 90 patients were randomly assigned to the control group or the CHGZ group. The patients in the CHGZ group received conventional Western medicine and CHGZ granules for 4 weeks, while the patients in the placebo group received conventional Western medicine and placebo. The efficacy for angina pectoris, nitroglycerin discontinuation rate, traditional Chinese medicine (TCM) syndrome score, seattle angina questionnaire (SAQ) score, cardiac markers, vascular endothelial function and safety indicators were evaluated before and after the intervention.

**Results:**

At the end of this study, compared with those in the control group, the curative effect on angina pectoris; the total nitroglycerin discontinuation rate; the total TCM syndrome score; and the scores of several dimensions of the SAQ, B-type natriuretic peptide precursor (NT-proBNP), asymmetric dimethylarginine, endothelial nitric oxide synthase, nitric oxide (NO) and endothelin 1 in CHGZ group were significantly greater (*P* < .05 or *P* < .01).

**Conclusion:**

CHGZ may be a beneficial adjuvant treatment for SAP patients. CHGZ can effectively improve clinical symptoms, quality of life, and vascular endothelial function and is safe. The detailed mechanism through which CHGZ affects vascular endothelial function should be further studied in the future.

## 1. Introduction

Cardiovascular diseases (CVDs) are the leading cause of death worldwide,^[1,2]^ and coronary artery disease (CAD) has the highest mortality rate among CVDs.^[3]^ Stable angina pectoris (SAP) is a very common ischemic cardiovascular disease^[4,5]^ characterized by left anterior chest pain or discomfort in an adjacent area.^[6]^ The diagnosis and treatment of patients with CAD have improved to some extent in recent years,^[7,8]^ and most patients with SAP receive optimal pharmacologic therapy or interventional therapy when necessary^[9,10]^; however, these patients still experience a high number of clinical symptoms and a poor prognosis,^[11]^ and SAP has become a global public health problem.^[12]^ Therefore, there is an urgent need to develop new adjuvant therapies for these patients, such as TCM, for the treatment of SAP; these therapies can improve the quality of life and clinical symptoms of patients. The main pathological basis for the development of CAD is atherosclerosis^[13,14]^; vascular endothelial dysfunction is the initiating factor of atherosclerosis,^[15,16]^ and relevant indices of endothelial function impairment can be detected before clinically apparent lesions.^[17]^ The abnormal release of vasoactive substances during vascular endothelial cell injury can serve as a specific molecular marker^[18]^ that can help to assess the extent of vascular endothelial injury and provide a basis for early diagnosis, treatment, and prognostic evaluation of this disease.^[19,20]^

Studies have shown that Chinese herbal compounds such as musk heart pills and crescent capsules can be useful adjuncts to conventional treatment, significantly reducing the incidence of angina and the risk of cardiovascular events among patients with stable CAD^[21,22]^; however, clinical studies of Chinese herbal compounds as adjuncts in the treatment of patients with SAP are still relatively scarce, and the underlying mechanism needs to be further clarified. Chaihu Guizhi decoction is derived from the classic Chinese herbal formula recorded in the Treatise on Typhoid Miscellaneous Diseases,^[23]^ which has been used in clinical practice for nearly 1700 years and has been shown to have good efficacy in treating respiratory, digestive, and cardiovascular diseases.^[24–26]^ The Chaihu Guizhi formula (CHGZ) used in the present study were generated from the original Chaihu Guizhi decoction formula supplemented with *Salvia miltiorrhiza* and Xianhe Cao, which are more suitable for the treatment of SAP. Excitingly, preliminary studies have demonstrated that some of the drugs used for CHGZ can improve clinical symptoms in patients with SAP^[27]^ and that different compounds used for CHGZ can improve vascular endothelial diastolic function.^[28–30]^ However, to date, no double-blind, placebo-controlled study on the use of CHGZ in patients with SAP or clinical study on the improvement of vascular endothelial function with CHGZ in patients with SAP has been performed.

Therefore, this study hypothesized that CHGZ would protect the vascular endothelium, relieve angina symptoms, and improve quality of life in SAP patients. To the best of our knowledge, this clinical trial is the first to examine the effect of adding CHGZ to conventional Western drug therapy on clinical outcomes and indicators of endothelial diastolic function in SAP patients.

## 2. Methods

### 2.1. Study design

This study was designed as a randomized, double-blind, placebo-controlled clinical trial with 2 parallel intervention and control groups. The study protocol was reviewed and approved by the Institutional Review Board of Yueyang Hospital of Integrative Medicine, Shanghai University of Traditional Chinese Medicine (2020-204), and registered in the China Clinical Trial Registry (ChiCTR2200065514). All participants provided written consent. This clinical trial followed the Consolidated Standards of Reporting Trials 2010 guidelines for reporting randomized trials.^[31]^

### 2.2. Participants

Participants with SAP were recruited and screened from December 2020 to July 2022 at the Department of Cardiology, Yueyang Hospital of Integrative Medicine, Shanghai University of Traditional Chinese Medicine (Shanghai, China). Participants who met the trial eligibility criteria were recruited mainly through recruitment announcements on posters and WeChat campaigns.

The diagnosis of SAP was in accordance with the European Society of Cardiology Guidelines for the Management of Stable Coronary Artery Disease, published by the European Society of Cardiology in 2013.^[4]^ The inclusion criteria were as follows: were diagnosed with the diagnostic criterion of SAP of CAD and were clinically stable within 4 weeks before randomization to the group; met the diagnostic criteria for chest paralysis related to qi stagnation and cardiothoracic syndrome; aged 40 to 80 years; had not taken part in any organized Chinese medicine treatment in the past 3 months; and voluntarily participated in this trial, signed an informed consent form and were able to cooperate with the treatment.

The exclusion criteria were as follows: acute exacerbation requiring a change in medication or hospitalization; coexistence of other acute cardiovascular diseases; pregnancy, lactating women, or women with childbearing requirements; combination of liver, kidney, hematopoietic system, and endocrine system and other serious illnesses; allergy or multidrug allergy; and current participation in any experimental trials.

### 2.3. Sample size

The sample size was determined on the basis of angina efficacy based on the results of previous studies,^[32]^ and a two-sided test was performed at the α = 0.05 level, with a test efficacy of 0.8 and a final β = 0.2. Based on the sample size formula for the comparison of two-sample rates for count data, the minimum sample size required was calculated to be approximately 37 patients per group, and the final sample size was considered to be 45 patients per group, accounting for a 20% dropout rate.

### 2.4. Randomization and allocation concealment

A statistician who was unaware of the conduct and evaluation of this study randomized the patients. This was based on a pre-generated 90-person (1:1) randomization table created using IBM SPSS® version 26.0 (IBM Corp., Armonk). Based on the randomization table, each patient’s allocation was placed in an opaque envelope, sealed in a double envelope, and stored until the end of this study. The number of randomizations was communicated to the IP custodian who marked the IP on the label without differentiating by group. Participants who provided written consent and met all inclusion criteria were enrolled and assigned a randomization number by the Clinical Research Coordinator in the order of their visit.

### 2.5. Intervention

Participants were randomly assigned to receive the prescribed study medication. The CHGZ group received herbal CHGZ granules, and the Control group received placebo granules (1 bag of 3 g twice daily, 30 minutes after meals), for 4 weeks. Along with the intervention, all patients received conventional prescription medications, including Aspirin Enteric-coated tablets (100 mg, Bayer S.pA), Atorvastatin Calcium Tablets (20 mg, Huizhi Pharmaceutical Company), according to the 2018 Guidelines for the Rational Use of Medications in Coronary Heart Disease.^[33]^

### 2.6. Preparation of placebo and CHGZ granules

#### 2.6.1. CHGZ granules

The ingredients of each bag of CHGZ extract granules included 12 g of Chai Hu, 5 g of Gui Zhi, 5 g of Paeoniae Alba, 6 g of Semen Heterophyllum, 5 g of Scutellariae Scutellariae, 5 g of Panax Ginseng, 3 g of roasted Glycyrrhiza, 5 g of ginger, 6 g of jujubes, 15 g of Xianhe Cao, and 12 g of Dangshen (Danshen). A total of 3.0 g of brown CHGZ extract was obtained by mixing the dried CHGZ extract (1.56 g) and 1.44 g of maize starch and drying them to form granules.

#### 2.6.2. Placebo granules

The placebo granules were formulated with color, odor, and taste in mind to minimize external differences between participant and investigator blinding and the actual medication. Optimal prescriptions were determined by performing disintegration tests to determine the stability of the granules during storage and examining their characteristics. The placebo granules (3.0 g/bag) were composed of 1/20th of the active ingredients in the CHGZ pellets, corn starch and cocoa coloring.

Both CHGZ and placebo pellets were hermetically sealed and packaged in opaque containers with identical labels; both were prepared by Jiangyin Tianjiang Pharmaceutical Co., Ltd., in China under production lot number H20051499, and both were manufactured according to the Chinese Good Manufacturing Practice guidelines. All pellets were stored at room temperature (below 25°C) in a dry and light-protected place before the start of this study.

### 2.7. Medication adherence assessment

Participants in each group received pellets at Visit 1 (Week 0) and Visit 2 (Week 3). The investigator checked the amount of medication remaining to ensure that the participant was taking the medication as directed (Visit 2). Medication adherence (%) was calculated every 2 weeks by dividing the number of medications actually taken by the number of medications that should have been taken, with a threshold of 80% for efficacy analysis. If medication adherence was <80%, the participant was excluded from the protocol compliance analysis.

### 2.8. Blinding

This trial was double-blinded by generating CHGZ pellets and placebo pellets that were externally indistinguishable for the CHGZ and control groups, respectively. The manufacturer packed the IP packages identically to hide intervention allocation and affixed randomization numbers to the IP containers as well as the placebo according to a predesigned randomization table to ensure double blinding. Participants, investigators, pharmacists, and outcome assessors did not know which container contained the study drug or placebo, nor did they know the randomization assignment of patients.

### 2.9. Outcomes

Outcome measures were evaluated by a board-certified cardiovascular physician who was unaware of the participants’ group assignment. Assessments were performed at baseline and after completion of the intervention (4 weeks).

### 2.10. Angina efficacy

The clinical efficacy of the control group was compared with that of the CHGZ group according to the “Coronary heart disease angina pectoris and electrocardiogram efficacy evaluation standard.”^[34]^ The apparent effects were as follows: Grade I or II or mild or moderate angina symptoms disappeared or basically disappeared without nitroglycerin; original Grade III or greater severe angina attacks compared with a pretreatment reduction of more than 2 grades; after treatment, the severity of angina and duration and frequency of angina significantly decreased; class II, III or moderate, angina was more severe than before treatment, and the angina grade was reduced by 1 level or 1 degree; and if there was no change in the degree of angina attacks compared with that before treatment, the treatment was ineffective; and increase of 1 grade or more compared with the angina attack before treatment indicated aggravation.

### 2.11. Discontinuation rate of nitroglycerin

According to the “Guidelines for Clinical Research of New Chinese Medicines,” the discontinuation rate was calculated as follows: discontinuation rate = [(number of tablets before treatment – number of tablets after treatment)/number of tablets before treatment] × 100%. The discontinuation rate of nitroglycerin was compared between the control group and the CHGZ group. The rates of nitroglycerin reduction and discontinuation in the control group and CHGZ group were as follows: discontinuation: patients stopped using nitroglycerin completely after treatment; dosage reduction: patients’ dosage of nitroglycerin was reduced by more than 50% after treatment; no change: patients’ dosage of nitroglycerin was reduced by <50% after treatment or remained unchanged; and dosage after treatment was increased compared with that before treatment.

### 2.12. Evaluation criteria for Chinese medicine evidence points

The CHGZ group and the control group were scored according to the Guidelines for Clinical Research of New Chinese Medicines. The scoring method was as follows: TCM syndromes were divided into 4 categories: none, mild, moderate and severe. The primary symptoms were chest pain, chest tightness, and chest distension, which were quantified as 0, 2, 4, and 6 points, respectively, according to the severity of the symptoms. The secondary symptoms were distention and stuffiness in the epigastrium, palpitations, irritability, and insomnia; these symptoms were quantified as 0, 1, 2, or 3 points depending on the severity of the symptoms. Severe symptoms included a dark red tongue, thin or greasy tongue coating, and thin and stringy pulse.

### 2.13. Seattle angina questionnaire (SAQ)

The seattle angina questionnaire (SAQ) was used to assess the quality of life of patients with coronary angina in 5 dimensions: the degree of physical activity limitation (PL), the angina steady state (AS), the angina attack frequency (AF), treatment satisfaction (TS), and disease perception (DP).^[35]^ The total score was 100, with higher scores indicating better quality of life.

### 2.14. Blood biomarker testing

Fasting venous blood samples (8 hours after the last meal) were collected by trained medical staff from Yangzhou Hospital of Traditional Chinese Medicine Affiliated to Shanghai University of Traditional Chinese Medicine. The levels of endothelial nitric oxide synthase (eNOS) (EH6595S), asymmetric dimethylarginine (ADMA) (EH6913S), and endothelin 1 (ET-1) (EH6910S) were determined using the ELISA method, with the ELISA instrument DG5033A from Huadong Electronics Co., Ltd. (Nanjing, China). The endothelial-derived nitric oxide (NO) was measured using the nitrate reductase method (No. A12-1-1, ZD-6A fully automatic nitrate reductase instrument, Beijing, China). Plasma samples before and after the intervention were stored at −80°C in the refrigerator before testing, and the samples were analyzed immediately after thawing. High-sensitivity troponin T (HsTnT) and B-type natriuretic peptide precursor (NT-proBNP) are tested by the Laboratory Department of the Yueyang Hospital of Integrated Traditional and Western Medicine, affiliated with Shanghai University of Traditional Chinese Medicine.

### 2.15. Safety assessment

Each participant’s medical history was recorded at the initial screening visit. By conducting weekly in-person or telephone follow-ups to monitor participants’ clinical symptoms, and performing clinical laboratory tests at baseline and after completion of the intervention (4 weeks), the safety of the CHGZ intervention for stable angina was assessed.

### 2.16. Statistical analysis

The data were analyzed using SPSS version 26.0 software (IBM Corporation, Armonk). Continuous variables were evaluated using tests of normality and homogeneity of variance. Variables are presented as the means and standard deviations, and intra- and intergroup comparisons were performed by paired *t* tests and independent *t* tests, respectively. Variables are described using medians and interquartile ranges (M(Q25, Q75)) if the data did not conform to a normal distribution, and within- and between-group comparisons were performed by Wilcoxon and Mann–Whitney *U* tests, respectively. Categorical variables were tested using χ^2^ tests, and variables are presented as frequencies (expressed as percentages). A *P*-value of <.05 indicated statistical significance.

## 3. Results

### 3.1. Participant characteristics

**Figure 1.**
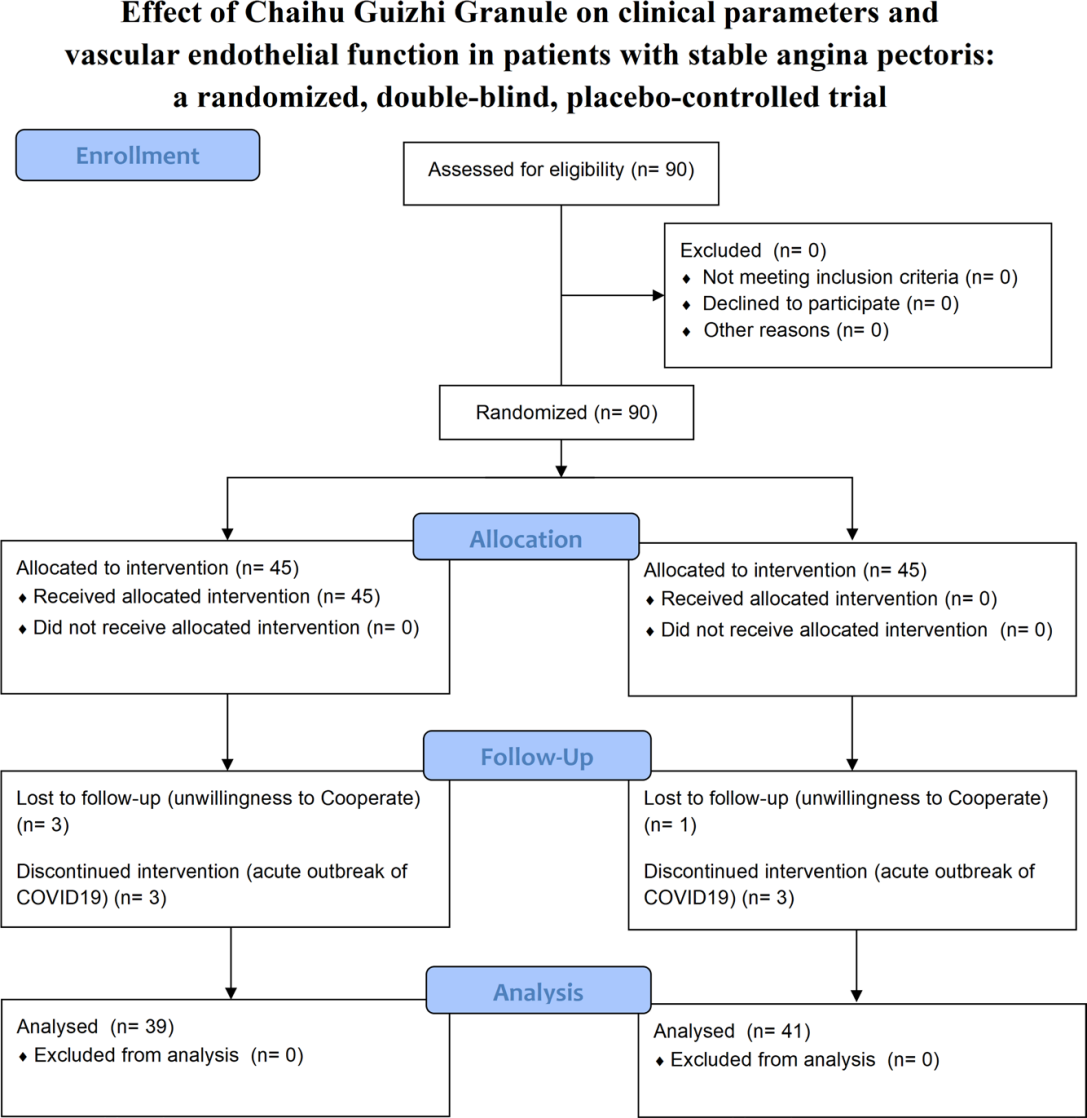
Flowchart of the trial design.

A total of 90 eligible participants were recruited from December 2020 to July 2022 for this study. Finally, 80 patients (CHGZ group = 41, control group = 39) completed this clinical trial (Fig. 1). Ten participants were excluded from the study for the following reasons: 6 cases of sudden infection with Coronavirus disease 2019, 3 from the control group and 3 from the CHGZ group; 4 cases of loss to follow-up (3 from the control group and 1 from the CHGZ group). The characteristics of all participants, including age, sex, angina classification, disease duration, smoking status, and comorbidities, are shown in Table 1. There was no statistically significant difference between the 2 groups in terms of characteristics (*P* > .05; Table 1).

**Table 1:** Baseline demographic characteristics of subjects in the CHGZ and control groups.

Characteristics	Control (n = 39)	CHGZ (n = 41)	*P*-value
Age (yr)	63.15 ± 7.16	63.0 ± 6.34	.919*
Gender (male/female, n%)	22 (56.4%)/ 17 (43.6%)	27 (65.9%)/ 14 (31.1%)	.386†
Angina pectoris grading, n (%)			
I	13	18	.608‡
II	22	20	
III	4	3	
Duration of illness (mo)	36 (24, 60)	36 (24, 72)	.827‡
Smoking, n (%)	13 (33.3)	15 (36.6)	.761†
Comorbidities, n (%)			
High blood pressure	23 (59.0)	27 (65.9)	.489‡
Diabetes	22 (56.4)	16 (39.0)	
High blood fat disease	13 (33.3)	12 (29.3)	

Values are expressed as mean ± standard deviation or median and interquartile spacing M (Q25, Q75) or n%.

CHGZ = Chai Hu Gui Zhi Tang.

* Comparisons between groups (independent *t*-test and Mann–Whitney *U*-test)

† Comparisons between groups (χ^2^ test).

‡ Comparisons between groups (Mann–Whitney *U*-test).

### 3.2. Effect of the CHGZ intervention on patient outcomes

#### 3.2.1. Primary efficacy outcome

##### 3.2.1.1. Angina efficacy

As shown in Table 2, the total angina efficacy rate in the treatment group was as high as 82.9%, which was significantly better than that of 61.5% in the control group (*P* < .05; Table 2). Among those with good angina efficacy in the control group, 15 (38.5%) had ineffective treatment, 18 (46.2%) had effective treatment, and 6 (15.4%) had obvious efficacy; however, among those with angina efficacy in the CHGZ group, 7 (17.1%) had ineffective treatment, 20 (48.8%) had effective treatment, and 14 (34.1%) had obvious efficacy.

**Table 2:** Comparison of angina efficacy and nitroglycerin reduction rate between the 2 groups before and after intervention.

Outcome variables	Control (n = 39)	CHGZ (n = 41)	*P*-value
Angina efficacy			
Overall effectiveness, n (%)	24 (61.5)	34 (82.9)	.032
Obvious effective, n (%)	6 (15.4)	14 (34.1)	
Effective, n (%)	18 (46.2)	20 (48.8)	
Ineffective, n (%)	15 (38.5)	7 (17.1)	
Nitroglycerin remission rate			
Total discontinuation rate, n (%)	21 (52.8)	32 (78.0)	.022
Discontinuation n (%)	6 (15.4)	12 (29.3)	
Reduction n (%)	16 (41.0)	20 (48.8)	
No change n (%)	17 (43.6)	9 (22.0)	

Values are presented as numbers (%); *P*-value for the between-group comparison using Pearson χ^2^ test.

#### 3.2.2. Secondary efficacy outcomes

##### 3.2.2.1. Comparison of the nitroglycerin discontinuation rate

As shown in Table 2, the total nitroglycerin reduction and discontinuation rate in the CHGZ group was as high as 78.0%, which was significantly better than that of 52.8% in the control group (*P* < .05; Table 3). In the control group, 17 (43.6%) patients had an unchanged nitroglycerin rate, 16 (41.0%) had a reduction in nitroglycerin, and 6 (15.4%) discontinued nitroglycerin; in the CHGZ group, 9 (22.0%) had an unchanged nitroglycerin rate, 20 (48.8%) had a reduction, and 12 (29.3%) discontinued nitroglycerin.

**Table 3:** Comparison of TCM evidence points and SAQ scale points between the 2 groups before and after intervention.

Outcome variables	Measurement period	Control (n = 39)	CHGZ (n = 41)	*P* ^a^
Chinese medicine score	Before intervention	12 (8, 9)	14 (11, 15)	.505
	After intervention	6 (4, 8)**	5 (4, 6)**,***	.030
	*P* ^b^	<.001	<.001	
Degree of activity limitation	Before intervention	40 (32.22, 48.89)	37.78 (31.11, 46.67)	.633
	After intervention	42.22 (34.45, 51.11)**	42.22 (34.45, 51.11)**	.21
	*P* ^b^	<.001	<.001	
Stable state of angina pectoris	Before intervention	50 (25, 50)	25 (25, 50)	.569
	After intervention	75 (50, 75)**	100 (50, 100)**,****	.009
	*P* ^b^	<.001	<.001	
Episodes of angina pectoris	Before intervention	50 (30, 70)	50 (40, 60)	.899
	After intervention	60 (50, 80)**	80 (70, 80)**,***	.023
	*P* ^b^	<.001	<.001	
Treatment satisfaction	Before intervention	48.56 (28.56, 66.78)	33.89 (14.06, 64.89)	.235
	After intervention	57.89 (50, 68.42)*	78.95 (73.68, 89.47)**,****	<.001
	*P* ^b^	.034	<.001	
Degree of disease awareness	Before intervention	50 (41.67, 62.5)	50 (41.67, 58.33)	.754
	After intervention	58.33 (50, 66.67)**	66.67 (58.33, 75)**,****	.004
	*P* ^b^	<.001	<.001	

Distribution of the variable was not normal; Values are expressed as interquartile spacing M (Q25, Q75); *P*^a^ intergroup comparisons (Mann–Whitney *U* test); *P*^b^ intragroup comparisons (Wilcoxon rank sum test).

CHGZ = Chai Hu Gui Zhi Tang.

* *P* < .05 after treatment versus before treatment.

** *P* < .01 after treatment versus before treatment.

*** *P* < .05 CHGZ versus control.

**** *P* < .01 CHGZ versus control.

##### 3.2.2.2. Comparison of TCM evidence points

At the end of this study, intergroup comparisons revealed that the difference in total TCM symptom scores between the CHGZ group and the control group was statistically significant (*P* < .05; Table 4). Intragroup comparisons of the CHGZ group and intragroup comparisons of the control group revealed that the difference in total TCM symptom scores was statistically significant (*P* < .001; Table 3).

**Table 4:** Comparison of the levels of cardiac markers indexes in the 2 groups before and after intervention.

Outcome variables	Measurement period	Control (n = 39)	CHGZ (n = 41)	*P* ^a^
HsTnT (ng/mL)	Before intervention	0.01 ± 0.00	0.01 ± 0.00	.77
	After intervention	0.01 ± 0.00	0.01 ± 0.00	.96
	*P* ^b^	.79	.88	
NT-proBNP (pg/mL)	Before intervention	131.89 ± 20.26	128.74 ± 27.41	.56
	After intervention	129.22 ± 16.30	114.80 ± 18.37*,**	<.001
	*P* ^b^	.06	<.001	

Values are expressed as mean ± standard deviation; *P*^a^ intergroup comparisons (independent *t*-test); *P*^b^ intragroup comparisons (paired *t*-test).

CHGZ = Chai Hu Gui Zhi Tang, HsTnT = high-sensitivity troponin T, NT-proBNP = B-type natriuretic peptide precursor.

* *P* < .01 after treatment versus before treatment.

** *P* < .01 CHGZ versus control.

##### 3.2.2.3. Comparison of seattle angina questionnaire (SAQ) scores

At the end of this study, the results of the intergroup comparison showed that the improvements in the angina stable state (AS), angina attack frequency (AF), treatment satisfaction (TS), and disease perception (DP) scores of the CHGZ group were significantly greater than those of the control group (*P* < .05 or *P* < .01; Table 3), but the differences in the degree of physical activity limitation (PL) were not statistically significant (*P* > .05; Table 3).

The results of the within-group comparison in the control group showed statistically significant differences in the degree of physical activity limitation (PL), angina stable state (AS), angina attack frequency (AF), treatment satisfaction (TS), and disease perception (DP) (*P* < .05 or *P* < .01; Table 3). In addition, the results of intragroup comparisons of the CHGZ group showed that the differences in the above 5 dimensions of the SAQ were statistically significant (*P* < .01; Table 3).

##### 3.2.2.4. Comparison of HsTnT and NT-proBNP

At the conclusion of this study, the results of the intergroup comparison indicated a statistically significant difference between the CHGZ group and the control group in terms of improvement in NT-proBNP (*P* < .01; Table 4). However, there was no statistically significant difference between the CHGZ group and the control group in terms of improvement in HsTnT (*P* > .05; Table 4).

Intragroup comparisons within the control group showed no statistically significant difference in HsTnT (*P* > .05; Table 4); no statistically significant difference in NT-proBNP (*P* > .05; Table 4). Additionally, the intra-group comparison analysis in the CHGZ group showed that the difference in HsTnT was not statistically significant (*P* > .05; Table 4); the difference in NT-proBNP was statistically significant (*P* < .01; Table 4).

##### 3.2.2.5. Comparison of ADMA, eNOS, NO and ET-1

At the end of this study, the results of the between-group comparison revealed statistically significant differences in the improvements in ADMA, eNOS, NO and ET-1 in the CHGZ group compared with those in the control group (*P* < .05 or *P* < .01; Table 5).

**Table 5:** Comparison of the levels of vascular endothelial diastolic function indexes in the 2 groups before and after intervention.

Outcome variables	Measurement period	Control (n = 39)	CHGZ (n = 41)	*P* ^a^
ADMA (ng/mL)	Before intervention	40.19 ± 13.42	38.40 ± 13.39	.553
	After intervention	35.72 ± 10.80*	27.85 ± 9.03**,****	.001
	*P* ^b^	.014	<.001	
eNOS (pg/mL)	Before intervention	291.42 ± 130.20	297.02 ± 145.42	.857
	After intervention	323.96 ± 160.25	418.72 ± 190.37**,***	.019
	*P* ^b^	.326	<.001	
NO (µmol/L)	Before intervention	26.41 ± 15.28	25.56 ± 14.94	.801
	After intervention	28.11 ± 13.79	36.48 ± 21.72**,***	.043
	*P* ^b^	.279	<.001	
ET-1 (pg/mL)	Before intervention	78.81 ± 23.57	80.77 ± 28.48	.739
	After intervention	69.90 ± 22.36*	58.34 ± 20.32**,***	.018
	*P* ^b^	.024	58.34 ± 20.32**,***	

Values are expressed as mean ± standard deviation; *P*^a^ intergroup comparisons (independent *t*-test); *P*^b^ intragroup comparisons (paired *t*-test).

ADMA = asymmetric dimethylarginine, eNOS = endothelial nitric oxide synthase, ET-1 = endothelin 1, CHGZ = Chai Hu Gui Zhi Tang, NO = nitric oxide. *P < .05 after treatment versus before treatment.

** *P* < .01 after treatment versus before treatment.

*** *P* < .05 CHGZ versus control.

**** *P* < .01 CHGZ versus control.

The results of within-group comparisons in the control group showed that the differences in ADMA and ET-1 were statistically significant (*P* < .05; Table 5); the differences in eNOS and NO were not statistically significant (*P* > .05; Table 5). In addition, intragroup comparative analysis of the CHGZ group revealed statistically significant differences in ADMA, eNOS, NO and ET-1 (*P* < .01; Table 5).

### 3.3. Adverse events and safety

In the control group, there was one case of mild nausea and one case of diarrhea; in the CHGZ group, there was one case of mild diarrhea. After adjusting the duration and method of drug administration, the patients’ symptoms improved, Therefore, they did not withdraw from this study. Six cases of sudden infection with coronavirus disease 2019 (3 from the control group and 3 from the CHGZ group), And they withdrew from this study. No other adverse events were observed. There were no statistically significant differences in laboratory test results for routine blood parameters (morning fasting glucose, aspartate aminotransferase, alanine aminotransferase, blood urea nitrogen, creatinine, sodium, potassium, chloride, lactate dehydrogenase, or creatine phosphokinase) or electrocardiogram results before and after treatment.

## 4. Discussion

This was a randomized, double-blind, placebo-controlled clinical trial evaluating the efficacy of CHGZ adjunctive therapy in patients with SAP. The results of the present study support our hypothesis that twice-daily uninterrupted CHGZ treatment for 4 weeks improves angina symptoms and quality of life, modulates endothelial diastolic contractile function, and is a safe and effective adjunctive therapy in patients with SAP. This new study provides the first evidence to support the therapeutic potential of CHGZ as a treatment for SAP.

In recent years, progress has been made in treating coronary heart disease through modern drug therapy^[36,37]^ and hemodialysis,^[37–39]^ but there are still problems such as poor improvement in patients’ angina efficacy, low quality of life, side effects from drugs, and a low long-term survival rate.^[40]^ Herbal medicine, as valuable treatment from China, has a long history of effectiveness in the prevention and treatment of coronary heart disease.^[41–43]^ A large body of evidence suggests that endothelial dysfunction is a key factor in the progression of atherosclerosis and plaque,^[44]^ that endothelial function is an independent predictor of CAD,^[45]^ and that monitoring changes in vascular endothelial function and targeting interventions can reduce the prevalence of coronary heart disease and mortality. The abnormal release of vasoactive substances during vascular endothelial cell injury can be used as a specific molecular marker for assessing the extent of endothelial injury and provide a basis for early diagnosis, treatment and prognostic evaluation of this disease.^[46,47]^ Tracing the root cause of endothelial protection and improving endothelial function during clinical treatment provides a more favorable long-term prognosis for patients with CAD.^[48]^

Coronary heart disease is classified as “chest paralysis and heart pain” in traditional Chinese medicine (TCM),^[49]^ and Chinese herbs are useful adjunctive therapies for the treatment of coronary heart disease. CHGZ is a famous formula in the Treatise on Miscellaneous Diseases of Typhoid Fever of the Medical Sage Zhang Zhongjing,^[24]^ and CHGZ can improve sudden cardiac and abdominal pain. Previous clinical experience has confirmed the good clinical efficacy of CHGZ in the treatment of coronary heart disease.^[27]^ CHGZ consists of Chai Hu, Scutellaria baicalensis, Gui Zhi, Paeonia lactiflora, Radix ginseng, Semen Xia, ginger, jujubes, and liquorice.^[23]^ Several scholars have conducted experimental studies on the extracts of single Chinese herbs. Chaihu can effectively prevent and control atherosclerosis, possibly through its ability to improve vascular endothelial function, improve cholesterol metabolism, and regulate blood coagulation and the fibrinolytic system.^[50,51]^ Cinnamyl alcohol can enhance coronary blood flow, thus exerting cardioprotective effects.^[52,53]^ Baicalin effectively improves endothelial dysfunction induced by myocardial ischemia/reperfusion injury^[29]^ and plays a protective role in many cardiovascular diseases, such as atherosclerosis.^[54–56]^ Total paeoniflorin glycosides can effectively inhibit and reverse myocardial ischemia/reperfusion injury-induced cardiomyocyte apoptosis.^[57]^ Ginsenosides can improve atherosclerosis by activating the eNOS-NO-cGMP signaling pathway to protect the vascular endothelium and diastole.^[58–60]^ Active ingredients in *Salvia miltiorrhiza* and ginger have also been shown to regulate vascular endothelial function and improve atherosclerosis.^[28,61,62]^ Therefore, in summary, the combination of different compounds in CHGZ may be beneficial for CAD patients by improving vascular endothelial diastolic function.

This study confirmed that the CHGZ group was superior to the control group in terms of increasing the levels of NOS and NO and decreasing the levels of ADMA and ET-1 (*P* < .05). Endothelial dysfunction is the initiating factor of atherosclerosis, and ADMA is an endogenous NOS inhibitor. By decreasing the level of ADMA, the inhibitory effect on NOS activity is weakened; thus, there is an increase in the level of NOS, which improves NO-dependent endothelium-mediated vasodilatation. Additionally, the production of the vasodilatory regulator NO is increased, which acts on the membrane of vascular smooth muscle cells, such that CHGZ protects the endothelium and improves endothelial function, providing a more favorable long-term prognosis for patients with CAD. However, the specific mechanism through which CHGZ protects the endothelium or reverses endothelial damage should be explored in further studies.

## 5. Limitations

The short follow-up period was a limitation of the current study. In future studies, long-term interventions and follow-up on the long-term efficacy of CHGZ are recommended. In addition, the mechanism by which CHGZ affects vascular endothelial diastolic contractile function in SAP patients requires further investigation.

## 6. Conclusion

This study demonstrated that CHGZ can effectively improve the clinical symptoms of qi stagnation in patients with SAP caused by CAD and improve the quality of life of patients, effectively improving indices related to vascular endothelial diastolic function; moreover, the clinical application of CHGZ is safe. Therefore, CHGZ may be a useful adjunctive therapy for these patients.

## Acknowledgments

The authors would like to thank the patients who participated in this study. In addition, the authors would like to thank the Center for Laboratory Medicine, Yueyang Hospital of Integrative Medicine, Shanghai University of Traditional Chinese Medicine, for providing technical support.

## Author contributions

**Conceptualization:** Yu Cao, Yan Shen.

**Data curation:** Yu Cao, Sihui Wang, Menghua He, Yan Shen.

**Funding acquisition:** Yan Shen.

**Methodology:** Yu Cao, Sihui Wang, Menghua He.

**Supervision:** Yan Shen.

**Writing – original draft:** Yu Cao.

**Writing – review & editing:** Yan Shen.
